# Event and Comment

**Published:** 1907-03-15

**Authors:** 


THE
### DENTAL REGISTER.

Vol. LXI. March 15, 1907. No. 3.

### EVENT AND COMMENT.

### Annual Clinic of the Detroit Dental Society.

Saturday February 16th, the Detroit Dental Society surpassed its former record in holding one of the best meetings in its history. The clinics were begun at ten o'clock in the morning, and were kept up until four in the afternoon. About fifty clinics were presented, and judging from the interest manifested by large attendance and discussions on every side, they were greatly appreciated. The clinics were largely of the "table" variety and an innovation consisted in having the clinicians on platforms two feet above the floor, so that the spectators could not disturb the clinicians by crowding around them, and as they passed along they could easily see everything that was displayed. The idea is an excellent one, for it gives all a chance to see and prevents a few from monopolizing the popular clinician. It also gives the clinician a chance to do his work to the best advantage.

Another popular idea was to have such chair clinics as could be so given on an elevated platform where all could see, and the demonstrator accompanied each with a short lecture or talk on what he would do. A considerable number of the clinics were given in this way and the scheme worked out very successfully.

At the close of the day Dr. V. H. Jackson of New York City gave an illustrated lecture on his method of correcting irregularities of the teeth, by what is known as the removable wire appliances. The lecture was well attended and very much enjoyed, as Dr. Jackson is an enthusiast and has accomplished remarkable results with very simple appliances. His claim is that his appliances are less painful to the patient, require less attention on the part of the dentist, are more easily kept clean and sanitary, and will produce as effective results as any other system or method. At seven o'clock a banquet was given in honor of the principal guests of the society: Dr. C. N. Johnson, of Chicago. at the Fellow Craft Club. It was not unlike other similar functions, except that it was nicer than most of them. The dinner was exceedingly delightful and the toasts and good fellowship eloquent and inspiring. Good speaking singing and eating took up so much time that Dr. Johnson who was to make the after dinner address was somewhat handicapped for time. But he spoke so earnestly and eloquently that he held his audience until it was too late for most of the out-of-town guests to get away that night. Take it all in all we have seldom attended a dental meeting which compassed so much in so short a space of time, and in which the work done was of so high a technical and professional character. The management deserves the greatest credit for this valuable stimulus to professional enthusiasm and attainment. We often wonder what more can be done to make dental society work more interesting and instructive. We are beginning to believe that the inventive genius of the younger men who are full of professional enthusiasms will find still more attractive methods of getting together, and we shall make still greater achievements in the days to come, if all who can will help a little.

**Taggart Cast Inlay.**

A new and most interesting process of casting high fusing metals has just been invented by Dr. Wm. Taggart, of Chicago. Dr. Taggarts invention was designed primarily for making cast gold inlays, but there seems no reason why it may not be extended to bridge work and plates as well. It will also find a wide application in all the applied sciences and arts. The method will be found described in our *Indents* section of this number of the Register. Specimens of the work were exhibited at the Detroit Clinic, and they certainly command confidence and respect for the method. The idea is to make a wax filling to fit the cavity exactly in form of contour and detail. This wax filling is removed from the cavity intact and inserted in a fine fire proof investment in a suitable metal flask which is a part of the casting apparatus. The flask is then heated and the wax being melted is absorbed by the investment leaving a matrix in which the casting is made. This is donc by melting pure gold by an oxyhydrogen blow pipe and quickly forcing it boiling hot into the matrix by an air pressure of thirty pounds and holding it there under this pressure until the gold chills, to prevent the shrinkage which would otherwise take place in the cooling process. The matrix investment is not heated very hot, consequently the pressure does not need to be maintained very long, probably from thirty to sixty seconds. The results are sharp and accurate casts that are of the same size and detail as the matrix used. The apparatus, while not specially complicated, like many other things of this sort, will need to be used skilfully, as there is no doubt but that the personal equation will here enter largely into the problem of the highest excellence as it does in other things. We predict it will meet with failure and poor results in the hands of the tyro the same as all other good things do. It may not bring the milenium of dental art; but it seems probable that another very useful process has been added to our technical armamentarium for which we should be exceeding grateful to a tireless and recognized expert in our technical art.

